# Effect of rehydration on guaiac-based faecal occult blood testing in colorectal cancer screening.

**DOI:** 10.1038/bjc.1993.210

**Published:** 1993-05

**Authors:** G. Castiglione, M. Biagini, A. Barchielli, G. Grazzini, A. Mazzotta, P. Salvadori, L. Scillone, S. Ciatto

**Affiliations:** Centro per lo Studio e la Prevenzione Oncologica, Florence, Italy.

## Abstract

Screening for colorectal cancer by means of unhydrated Hemoccult (HO) is in progress in the Province of Florence since 1982. In 1990 rehydrated HO was introduced in the town of Empoli. Five adjacent municipalities where screening had started in 1987 were selected for comparison. In both areas subjects aged 40-70 were invited by mail to undergo the screening protocol. HO-positive subjects were invited to undergo either pancolonoscopy or a combination of left colonoscopy and double contrast barium enema. HO-negative subjects were invited to repeat screening 2 years later. The positivity rate of HO was significantly higher (P < 0.001) for rehydrated (5%) as compared to unhydrated (3.1%) HO. The positive predictive values for cancer (unhydrated: 5.8%; rehydrated: 8.9%) and for adenomas (unhydrated: 26.7%; rehydrated: 25.5%) did not significantly differ. The detection rates of rehydrated HO were significantly higher as compared to unhydrated HO both for cancer (0.37% vs 0.15%; P < 0.05) and adenomas (1.06% vs 0.72%; P < 0.05%). In the present experience rehydration doesn't produce any decrease in the positive predictive value for cancer or adenomas and the increase in the positivity rate appears quite acceptable when considering the significant increase in the detection rates of cancer and adenomas. We conclude that rehydrated HO should be introduced as the standard test for screening in order to increase sensitivity for colorectal cancer and adenomas.


Br. J. Cancer (1993), 67, 1142–1144 © Macmillan Press Ltd., 1993

SHORT COMMUNICATION

### Effect of rehydration on guaiac-based faecal occult blood testing in colorectal cancer screening

G. Castiglione[1], M. Biagini[2], A. Barchielli[1], G. Grazzini[1], A. Mazzotta[1], P. Salvadori[3], L. Scillone[1] & S. Ciatto[1]

[1]*Centro per lo Studio e la Prevenzione Oncologica, Viale A. Volta 171, I-50131 Florence;* [2]*Endoscopy Unit, Ospedale Santa Verdiana, Via dei Mille 1, I-50051 Castelfiorentino;* [3]*Unità Sanitaria Locale N. 18, P.za XXIV Luglio 1, I-50053 Empoli, Italy.*

**Summary** Screening for colorectal cancer by means of unhydrated Hemoccult (HO) is in progress in the Province of Florence since 1982. In 1990 rehydrated HO was introduced in the town of Empoli. Five adjacent municipalities where screening had started in 1987 were selected for comparison. In both areas subjects aged 40–70 were invited by mail to undergo the screening protocol. HO-positive subjects were invited to undergo either pancolonoscopy or a combination of left colonoscopy and double contrast barium enema. HO-negative subjects were invited to repeat screening 2 years later.

The positivity rate of HO was significantly higher ($P < 0.001$) for rehydrated (5%) as compared to unhydrated (3.1%) HO. The positive predictive values for cancer (unhydrated: 5.8%; rehydrated: 8.9%) and for adenomas (unhydrated: 26.7%; rehydrated: 25.5%) did not significantly differ.

The detection rates of rehydrated HO were significantly higher as compared to unhydrated HO both for cancer (0.37% *vs* 0.15%; $P < 0.05$) and adenomas (1.06% *vs* 0.72%; $P < 0.05$ %).

In the present experience rehydration doesn't produce any decrease in the positive predictive value for cancer or adenomas and the increase in the positivity rate appears quite acceptable when considering the significant increase in the detection rates of cancer and adenomas.

We conclude that rehydrated HO should be introduced as the standard test for screening in order to increase sensitivity for colorectal cancer and adenomas.

Screening by faecal occult blood testing has been proposed to reduce colorectal cancer incidence and/or mortality. Hemoccult II (S.K.D.) (HO), based on guaiac impregnated cards, is the most common test used worldwide.

Evidence of screening efficacy is still lacking as the results of ongoing controlled studies are expected within a few years (Hardcastle *et al.*, 1989; Kronborg *et al.*, 1989; Mandel *et al.*, 1988).

Suboptimal estimates of sensitivity for colorectal cancer have been reported for HO, ranging from 50 to 70% (Hardcastle *et al.*, 1989; Kronborg *et al.*, 1989; Bertario *et al.*, 1988; Castiglione *et al.*, 1991). Rehydration of HO slides before development increases the sensitivity up to 85–90% (Kewenter *et al.*, 1988) but the consequent reduction in specificity with respect to unhydrated HO (from 98% to 95%) has been considered unacceptable for screening purposes and unhydrated HO is still employed in the majority of the ongoing experiences.

The aim of the present study was to compare the diagnostic accuracy of unhydrated and rehydrated HO for colorectal cancer and adenomas, as determined in two subgroups of subjects attending to a population based screening program.

### Materials and methods

A population-based screening for colorectal cancer by unhydrated HO has been in progress since 1982 in 24 municipalities of the Province of Florence. All subjects aged 40–70 living in the screening area have been invited every other year to undergo the screening protocol, run by the Centro per lo Studio e la Prevenzione Oncologica (CSPO) of Florence. Rehydrated HO was tested in the municipality of Empoli where screening started in September 1990. Five municipalities adjacent to the town of Empoli, where screening by unhydrated HO had been ongoing since 1987, were selected for comparison.

Colorectal cancer incidence and mortality in the two areas were comparable, according to the Tuscany Cancer Registry (Buiatti *et al.*, 1991).

In both areas responders were invited to collect faeces samples using HO kits on three consecutive bowel movements and advised not to eat rare red meat 2 days before and during faeces samples collection. Returned tests were developed in the CSPO laboratory in Florence, usually within 1 week from faeces samples collection.

The study was limited to faecal occult blood testing at first screening examination. Rehydrated HO was performed by 5,919 subjects from September 1990 to December 1991 (3,145 females, 2,774 males, mean age = 55.6, attendance rate = 36%), whereas unhydrated HO was performed by 7,129 subjects from April 1987 to December 1991 (3,131 females, 3,998 males; mean age = 53.6 years, attendance rate = 30%). No significant difference was evident between the two groups according to patients age and expected cancer incidence according to local Cancer Registry.

HO-negative subjects were advised to repeat screening after 2 years and to visit their family doctor to manage any complaint occurring during this period. Subjects with at least one positive HO determination were invited to undergo pancolonoscopy. Double contrast barium enema was undertaken when complete colonoscopy was not possible. Assessment was performed by experienced operators at the CSPO clinic and at the endoscopic unit of the screening area hospital.

The positivity rates, positive predictive values, and detection rates for cancer and adenomas were determined separately for unhydrated and rehydrated HO and then compared. Statistical significance of observed differences was set at a 0.05 $P$ level and checked by the chi-square test.

### Results

Table I reports the positivity rates and the detection rates of unhydrated or rehydrated HO and the compliance with the

Correspondence: G. Castiglione.
Received 14 August 1992; and in revised form 10 December 1992.

**Table I** Positivity rates and detection rates for cancer and adenomas of unhydrated and rehydrated HO, and compliance with diagnostic work-up in the two studied subgroups

| | *Unhydrated* | *Rehydrated* |
|---|---|---|
| Examined | 7,129 | 5,919 |
| HO-positive (positivity rate) | 218 (5.0%) | 291 (3.1%) |
| Assessed | 191 | 247 |
| Compliance with diagnostic work-up | 84.9% | 87.6% |
| Cancer patients (detection rate) | 11 (0.15%) | 22 (0.37%) |
| Adenoma patients (detection rate) | 51 (0.7%) | 63 (1.06%) |
| Largest adenoma | | |
| $>19$ mm (detection rate) | 18 (0.25%) | 23 (0.39%) |
| 10–19 mm (detection rate) | 26 (0.36%) | 23 (0.39%) |
| $<10$ mm (detection rate) | 7 (0.10%) | 17 (0.29%) |

**Table II** Diagnostic yield and positive predictive values (P.P.V.) for cancer and adenomas of unhydrated and rehydrated HO

| | *Unhydrated* | *Rehydrated* |
|---|---|---|
| Assessed | 191 | 247 |
| Cancer patients (P.P.V.) | 11 (5.8%) | 22 (8.9%) |
| Adenoma patients (P.P.V.) | 51 (26.7%) | 63 (25.5%) |
| Largest adenoma | | |
| $>19$ mm (P.P.V.) | 18 (9.4%) | 23 (9.3%) |
| 10–19 mm (P.P.V.) | 26 (13.6%) | 23 (9.3%) |
| $<10$ mm (P.P.V.) | 7 (3.7%) | 17 (6.9%) |

diagnostic work-up in the two studied subgroups. HO-positivity rate was significantly higher for rehydrated compared to unhydrated HO (5% *vs* 3.1%, chi-square = 29.8, df = 1, $P < 0.001$).

Significantly differences were evident between the detection rates of rehydrated and unhydrated HO both for cancer (0.37% *vs* 0.15% respectively, chi-square = 6.05, df = 1, $P < 0.05$) and adenomas (1.06% *vs* 0.72%, chi-square = 4.54, df = 1, $P < 0.05$). Statistical significance in adenoma detection rates was observed for smaller (10–19 mm: chi-square = 4.9, df = 1, $P < 0.05$; $< 10$ mm: chi-square = 6.3, df = 1, $P < 0.05$) and not for larger adenomas.

No significant difference was recorded in the two compared groups according to cancer site or stage, or adenoma size and correponding data are not reported.

Table II reports the positive predictive values (P.P.V.) of unhydrated and rehydrated HO for cancer and adenomas.

The P.P.V.s for cancer (unhydrated HO = 5.8%, rehydrated HO = 8.9%) or adenoma (unhydrated HO = 26.7%, rehydrated HO = 25.5%) in assessed subjects were not significantly different in the two compared groups. No significant difference was recorded in the two compared groups according to cancer site or stage and corresponding data are not reported.

### Discussion

The present study investigates the performance of unhydrated and rehydrated HO in two adjacent areas where a population based screening program is ongoing. Although the study is not randomised the comparability of the two subgroups is acceptable. In fact, cancer incidence and mortality in the two areas is similar, as obtained from the local cancer registry, and the age- and sex-adjusted expected incidence rates for colorectal cancer in the two compared subgroups was almost the same.

Symptomatic status of attenders was not recorded in this study, but screening modalities (recruitment methods, dietary restrictions before faecal samples collections) and assessment criteria were identical and attendance rates were low but consistent in both subgroups, thus excluding a major selection bias. Moreover the compliance of HO-positive subjects with the diagnostic work-up was similar in the two subgroups.

In our study unhydrated HO shows a higher positivity rate and a lower P.P.V. for cancer as compared to other population-based screening programs (Hardcastle *et al.*, 1989; Kronborg *et al.*, 1989). Both figures may be explained by the fact that the only dietary restriction in our recommendations for faecal occult blood testing concerns rare red meat in order to increase the acceptability of HO testing. This aspect needs to be reconsidered in the light of the introduction of rehydration (Macrae *et al.*, 1982). The lower P.P.V. for cancer compared to other screening programs could also be explained by the younger mean age of the asymptomatic screened population. In fact, our screening starts at age 40, in consideration of the earlier age of occurrence of adenomas as compared to cancer. This choice might be discussed on the basis of a cost/benefit analysis but this was not the purpose of this study.

Although the higher P.P.V. of rehydrated HO as compared to unhydrated HO (8.9% *vs* 5.4%; non-significant difference) is somewhat surprising, nevertheless the higher cancer and adenomas detection rates observed in the rehydrated with respect to the unhydrated HO subgroup, are more likely to be ascribed to a higher sensitivity of rehydrated HO rather than to a higher prevalence of colorectal neoplastic lesions. When rehydration was introduced we did not observe a parallel decrease in the positive predictive value for cancer or adenoma, as reported by other authors (Kewenter *et al.*, 1988). The increased recall rate to diagnostic assessment (5% *vs* 3.1%) with respect to unhydrated HO was quite acceptable in sight of the improved detection rate of cancer (0.37% *vs* 0.15%) and adenomas (1.06% *vs* 0.7%).

According to the results of the present study we believe that rehydrated HO should be introduced as the standard test for faecal occult blood testing in screening programs for colorectal cancer, as the reduction in specificity with respect to unhydrated HO seems to be outweighed by far by the improvement of sensitivity for both cancer and adenomas.

### References

BERTARIO, L., SPINELLI, P., GENNARI, L., SALA, P., PIZZETTI, P., SEVERINI, A., COZZI, G., BELLOMI, M. & BERRINO, F. (1988). Sensitivity of Hemoccult test for large bowel cancer in high-risk subjects. *Dig. Dis. Sci.*, **33**, 609–613.

BUIATTI, E., GEDDES, M., AMOROSI, A., BALZI, D., BARCHIELLI, A., BIGGERI, A., CARLI, S., CECCONI, R., GASPARI, R., SORSO, B. & VANNUCCHI, G. (1991). Cancer incidence and mortality in the Province of Florence. Quaderni di Oncologia, Firenze.

CASTIGLIONE, G., GRAZZINI, G., POLI, A., BONARDI, R. & CIATTO, S. (1991). Hemoccult sensitivity estimate in a screening program for colorectal cancer in the Province of Florence. *Tumori*, **77**, 243–245.

HARDCASTLE, J.D., CHAMBERLAIN, J., SHEFFIELD, J., BALFOUR, T.W., ARMITAGE, N.C., THOMAS, W.M., PYE, G., AMAR, S.S. & MOSS, S.M. (1989). Randomised, controlled trial of faecal occult blood screening for colorectal cancer. Results for first 107,349 subjects. *Lancet*, **1**, 1160–1164.

KEWENTER, J., BJOERK, S., HAGLIND, E., SMITH, L., SVANIK, J. & AHREN, C. (1988). Screening and rescreening for colorectal cancer. A controlled trial of fecal occult blood testing in 27,000 subjects. *Cancer*, **62**, 645–651.

KRONBORG, O., FENGER, C., OLSEN, J., BECH, O. & SONDERGAARD, O. (1989). Repeated screening for colorectal cancer with fecal occult blood test. A prospective randomized study at Funen, Denmark. *Scand. J. Gastroenterol.*, **24**, 599–606.

MACRAE, F.A., ST JOHN, D.J., CALIGIORE, P., TAYLOR, L.S. & LEGGE, J.W.K (1982). Optimal dietary conditions for Hemoccult testing. *Gastroenterology*, **82**, 899–903.

MANDEL, J.S., BOND, J.H., SNOVER, D., WILLIAMS, S., BRADLEY, M., WALKER, C., SCHUMAN, L.M. & GILBERTSEN, V. (1988). The University of Minnesota's Colon Cancer Control Study: Design and Progress to Date. In *Screening for Gastrointestinal Cancer*, Chamberlain, J. & Miller, A.B. (ed.) p. 17–24. Hans Huber Publishers: Toronto.


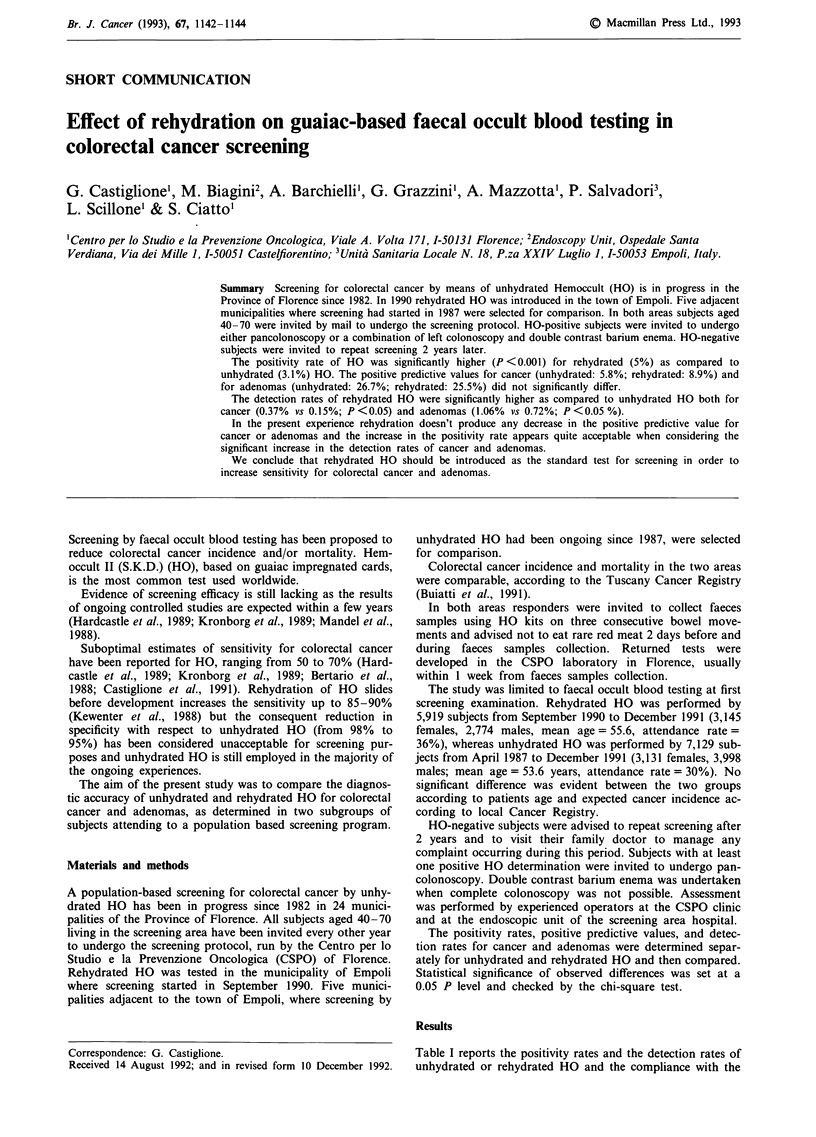

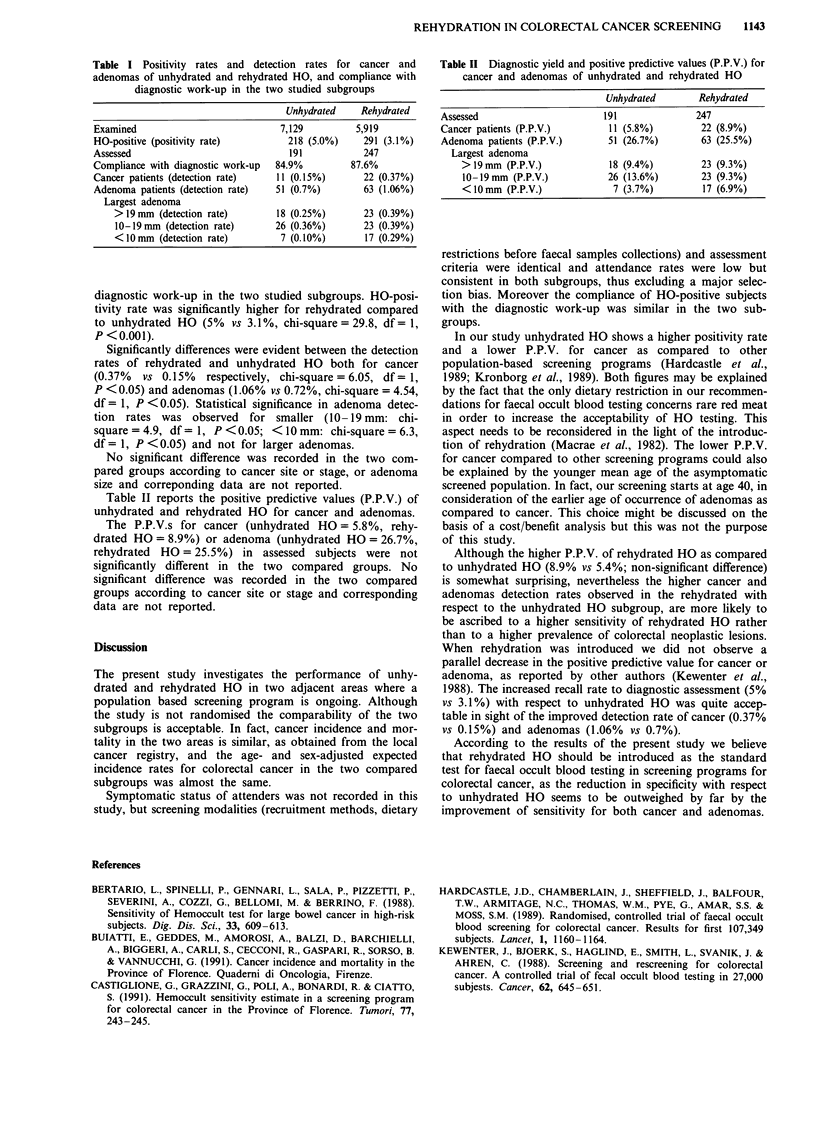

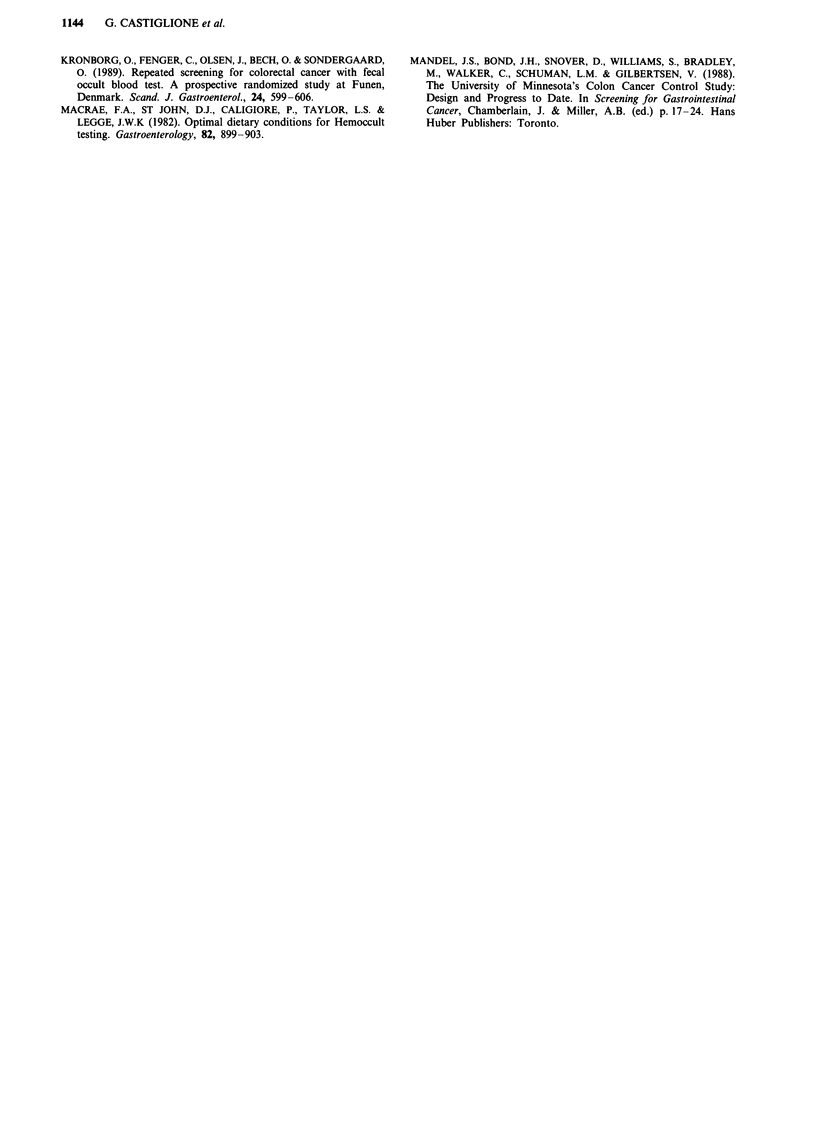


## References

[OCR_00260] Bertario L., Spinelli P., Gennari L., Sala P., Pizzetti P., Severini A., Cozzi G., Bellomi M., Berrino F. (1988). Sensitivity of Hemoccult test for large bowel cancer in high-risk subjects.. Dig Dis Sci.

[OCR_00272] Castiglione G., Grazzini G., Poli A., Bonardi R., Ciatto S. (1991). Hemoccult sensitivity estimate in a screening program for colorectal cancer in the Province of Florence.. Tumori.

[OCR_00278] Hardcastle J. D., Thomas W. M., Chamberlain J., Pye G., Sheffield J., James P. D., Balfour T. W., Amar S. S., Armitage N. C., Moss S. M. (1989). Randomised, controlled trial of faecal occult blood screening for colorectal cancer. Results for first 107,349 subjects.. Lancet.

[OCR_00285] Kewenter J., Björk S., Haglind E., Smith L., Svanvik J., Ahrén C. (1988). Screening and rescreening for colorectal cancer. A controlled trial of fecal occult blood testing in 27,700 subjects.. Cancer.

[OCR_00293] Kronborg O., Fenger C., Olsen J., Bech K., Søndergaard O. (1989). Repeated screening for colorectal cancer with fecal occult blood test. A prospective randomized study at Funen, Denmark.. Scand J Gastroenterol.

[OCR_00301] Macrae F. A., St John D. J., Caligiore P., Taylor L. S., Legge J. W. (1982). Optimal dietary conditions for hemoccult testing.. Gastroenterology.

